# What Do We Talk About Now When We Talk About Segmentectomy for GGO?

**DOI:** 10.3389/fsurg.2022.831246

**Published:** 2022-02-15

**Authors:** Hanyue Li, Chen Shen, Yang Chen, Yiyang Wang, Chenxi Zhong, Wentao Fang

**Affiliations:** Department of Thoracic Surgery, Shanghai Chest Hospital, Shanghai Jiao Tong University, Shanghai, China

**Keywords:** lobectomy, segmentectomy, ground glass opacity, early stage lung cancer, minimally invasive surgery

## Abstract

Segmentectomy has been considered as a compromised procedure in patients with early-stage lung cancer who could not tolerate standard lobectomy. By computed tomography (CT) screening, lung cancers are increasingly detected in earlier stages, especially those appearing as ground glass opacity (GGO)-containing lesions on CT scan. This has led to the revival of segmentectomy as an intentional procedure with the aim of curing selected patients, as GGO-containing lesions represent a special group of diseases that are relatively indolent in nature and seldom have lymphatic involvement. Limited resections, especially anatomical segmentectomy, may, thus, be helpful in reducing perioperative risks and preserving higher pulmonary function for patients while retaining similar oncological outcomes. However, clinical trials focusing specifically on the role of segmentectomy in the treatment of GGO-containing lung cancers are still lacking, especially in the minimally invasive surgery setting. Emerging evidence suggests that for such lesions, the oncological non-inferiority of segmentectomy to standard lobectomymay not be limited to lesions with a size ≤ 2 cm. More importantly, it is still unclear whether segmentectomy could indeed minimize perioperative risks and to what extent it could help preserve higher pulmonary function in good-risk patients with less extent of lung parenchyma resection. Hence, it is critical to reevaluate the efficacies of minimally invasive segmentectomy including not only oncological outcomes but also perioperative results and pulmonary function changes compared with lobectomy in good-risk patients with GGO-containing lung cancers. All these remain to be explored in future studies and robust evidence is still needed to prove that patients would indeed benefit from the combination of segmentectomy and minimally invasive surgery.

## Introduction

Segmentectomy was first introduced as an anatomical resection for lung cancer almost half a century ago (1). In 1995, the Lung Cancer Study Group (LCSG) trial (2) studied surgical, functional, and oncological results between lobectomy and limited resections in patients with stage Ia lung cancer. Compared to lobectomy, segmentectomy, together with wedge resection, was found to be associated with 75% increase in recurrence rate, 3 times more local recurrence, and significantly more overall cancer-related deaths, albeit better postoperative pulmonary function. Thereafter, lobectomy has been established as the standard procedure for early-stage non-small cell lung cancer, while segmentectomy is only considered as a palliative procedure for patients who are functionally unfit for lobectomy (2).

The LCSG trial was conducted almost three decades ago when there was no CT screening, minimally invasive surgery, or modern perioperative care. There have been huge changes in the scenario of thoracic surgery. Minimally invasive surgery (MIS), including both video-assisted thoracic surgery (VATS) and robotic surgery, has been shown as a safe procedure with limited risk of intraoperative bleeding, is associated with significantly diminished pain and trauma, and, therefore, better recovery and quality of life after surgery. Long-term outcomes, including survival and tumor recurrence, are equivalent to those of open thoracotomy (3, 4). With MIS now as a strongly recommended approach in clinical guidelines (5), it is necessary to reexamine its role together with limited resections in patients with early-stage lung cancer.

There has also been a significant change in disease profile, especially after the release of the National Lung Screening trial (6). Increasingly, smaller lesions have been detected, especially those appearing as ground-glass opacity (GGO)-containing nodules on CT scan. GGO lesions are more likely to represent a noninvasive histology (7), which is associated with low risk of lymphovascular invasion or nodal metastasis (8, 9). This has brought a second thought on limited resections, including segmentectomy, with a curative intent for these relatively indolent tumors. With growing evidence on segmentectomy's similar outcomes compare to those of standard lobectomy, sublobar resection is now accepted in clinical guidelines as an intentional procedure for a selected group of patients with early-stage lung cancer, in addition to it being a palliative surgery for high-risk patients (5).

Another problem with the LCSG trial is the mixture of segmentectomy with wedge resection in a sublobar group. In a Surveillance, Epidemiology, and End Results (SEER) database analysis, segmentectomy was associated with 20–30% survival advantage over wedge resection after propensity score matching (10). Thus, in the guidelines, segmentectomy is also recommended as a preferred procedure over wedge resection (5). Based on these concerns, this review article will focus specifically on segmentectomy for patients with GGO-containing early stage lung cancer regarding its oncological efficacy, perioperative risks, and pulmonary function impact, especially in the context of MIS.

## Oncological Outcomes After Segmentectomy in Patients With GGO-containing Lesions

Complete resection provides the best chance of cure for good-risk patients with early stage lung cancer. It is, thus, critically important to ensure first that segmentectomy is oncologically not inferior to lobectomy. Current indications for sublobar resections are limited to peripheral lesions ≤ 2 cm that are of pure AIS histology, GGO-dominant (≥ 50%) on CT scan, or have a long doubling time (≥ 400 days) (5). These correspond to AIS, MIA, and T1a tumors according to the 8th IASLC lung cancer staging system (11), in which the definition of T1 category has been changed from using the size of the total lesion to that of the solid component only. In fact, GGO-containing lesions may be the most suitable candidates for sublobar resections because of their indolent nature and less aggressive behavior (7). It has been reported that lesions containing a GGO component had less lymphatic invasion and less lymph node metastasis than solid nodules (8). The Japan Clinical Oncology Group (JCOG) 0804 trial focused on GGO-dominant (consolidation tumor ratio, CTR, ≤ 0.25) lesions ≤ 2 cm in size (12), corresponding to AIS and MIA in tumor stage and histology. Although over 80% of patients only underwent wedge resection, the 5-year overall and recurrence-free survival rates were both above 99%. No local recurrence was detected in this phase II study. Given that segmentectomy is technically much more demanding than wedge resection (13), the latter would be enough for AIS and MIA.

Two highly-anticipated phase III trials, JCOG 0802 and Cancer And Leukemia Group B (CALGB) 140503, both used tumor size ≤ 2 cm as their inclusion criteria. Patients with lymph node metastasis were excluded in both trials. CALGB 140503 is quite similar to the LGSG trial in study design, with the only exception that the upper limit of tumor size was reduced from 3 to 2 cm, to extrapolate the indication of sublobar resections to T1b tumors. There was still no discrimination between GGO-containing lesions and solid nodules, although it is now well-recognized that they are two different kinds of tumors by nature. Wedge resection was again grouped together with segmentectomy in the sublobar arm. If follow-up results could prove the non-inferiority of sublobar resection to lobectomy in survival and recurrence, it would mean that segmentectomy as a more extended procedure than wedge resection is oncologically appropriate for the more indolent GGO-containing lesions.

The JCOG 0802 trial has just released its initial follow-up outcomes (14). Focusing specifically on the results between segmentectomy and lobectomy in solid-dominant (CTR > 0.5) lesions ≤ 2 cm in size, it also aimed to extend the indication of segmentectomy to T1b tumors. It turned out that more than 50% of patients included actually had pure solid lesions (CTR = 1). Although it was designed as a non-inferior study, the overall survival after segmentectomy (94.3%) was actually superior to that of after lobectomy (91.1%, *p* = 0.008), with more deaths from other tumors found in the latter group. However, segmentectomy (10.5%) was still associated with significantly higher local recurrence rate compared to lobectomy (5.4%, *p* = 0.002) (14). The reason for this has not been revealed yet. It would, thus, be interesting to see if recurrence is associated with resection margin or CTR of lesions in segmentectomy patients.

**Figure 1:**
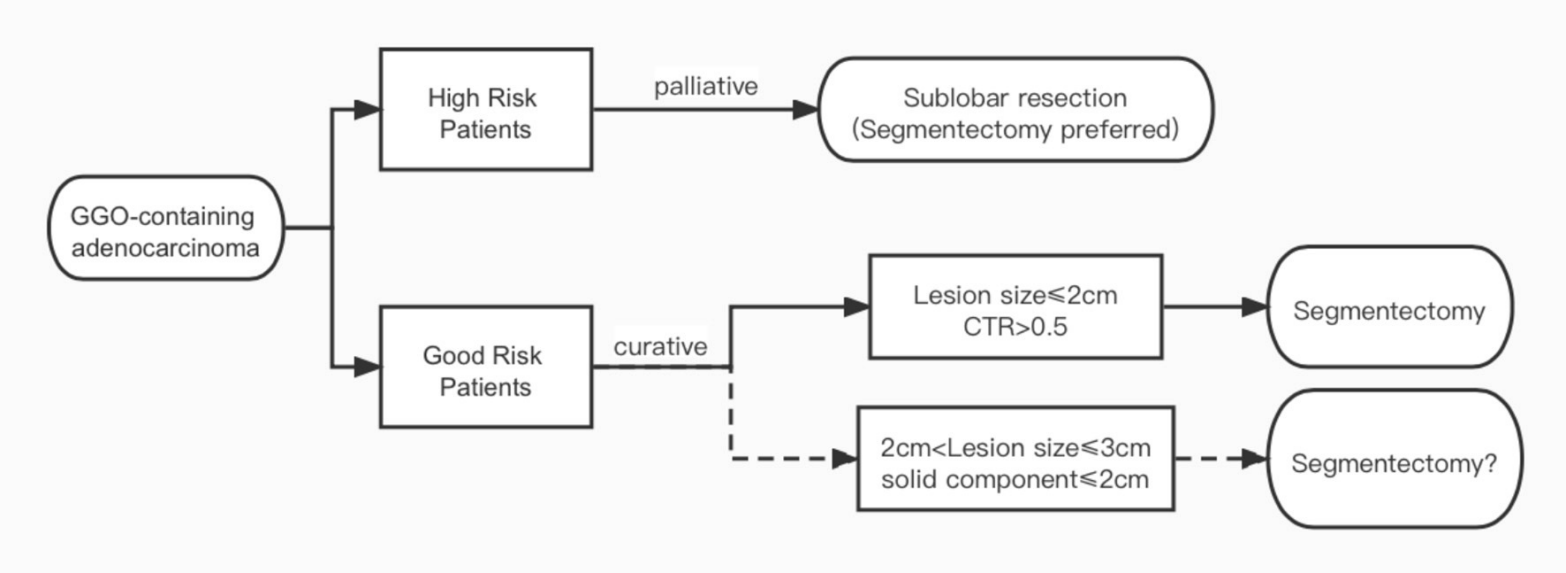
Indications of segmentectomy for GGO-containing early-stage lung cancers.

As mentioned above, the intention of the CALGB 140503 and JCOG 0802 trials was to extend the indication of segmentectomy to T1bN0M0 tumors. However, both trials used total lesion size as their inclusion criteria according to the 7th UICC staging, while in the 8th staging system released in 2016, the definition for tumor T category has been switched to using only solid component size in GGO-containing lesions (11). In a propensity score-matched study, Kamigaichiet al. (15) found similar recurrence-free survival rates between segmentectomy and lobectomy in both unmatched (82.7 vs. 73.4%, *p* = 0.3) and matched Japanese patients (80.1 vs. 79.5%) with radiologically solid dominant clinical stage Ia lung cancer measuring 2.1 to 3 cm. In another interesting large cohort study, Lin et al. (9) found that when matched by total lesion size, tumors appearing as solid dominant lesions on CT scan had significantly higher grade histology and worse survival than the GGO-dominant ones in Chinese patients. When matched by solid component size, however, GGO-containing tumors with different total lesion sizes actually had similar histology and outcomes regardless of GGO size. Even for patients with total lesion size of 2.1 to 3 cm, recurrence-free survival was still comparable after segmentectomy and lobectomy as long as solid component size was under 2 cm. Thus, it remains unclear whether segmentectomy would be oncologically equivalent to lobectomy specifically for GGO-containing lesions, especially in local recurrence. Special attention should be paid to lesions >2 cm but with a solid component size ≤ 2 cm to see if all T1b tumors could be completely resected by segmentectomy (Figure 1).

## Reducing Perioperative Risks in High-risk and Good-risk Patients

Sublobar resections have been recommended as an alternative to lobectomy for high-risk patients. The rationale is that there would be lower surgical risk with less extent of resection. Unfortunately there has been little evidence supporting this notion. In the LCSG trial, all 6 respiratory failures and 2 of the 3 postoperative deaths happened in the lobectomy group (2). Caution is needed when interpreting the results, as all patients underwent open thoracotomy in that study. The advent of MIS has greatly changed modern thoracic surgery. There has been ample evidence showing that compared to open procedures, MIS represented by VATS carries a significant advantage in reducing surgical trauma and postoperative pain, leading to better recovery and less surgical morbidity after lung cancer surgery (3). With trauma from incisions significantly reduced by MIS, surgical risks after pulmonary resections are now mostly decided by resection extent and complexity of the procedure *per se*.

In this concern, wedge resection is, without a doubt, much less traumatic than anatomical resections. It is a simple and straightforward procedure, in addition to having limited resection extent. Tsutani et al. found that patients who underwent wedge resection had significantly lower incidence of postoperative complications such as atelectasis and air leak than those who underwent segmentectomy (16). The perioperative results from the LCSG trial are not very helpful owing to the grouping of wedge and segmental resections together. In the safety analysis from the CALGB140503 trial with a similar study design, there were no statistically significant differences between lobectomy and sublobar resections in terms of perioperative mortality or overall morbidity (17). However, the rate of grades 3–5 complications was highest in segmentectomy patients (19%) than in lobectomy (16%) and wedge resection (11%) patients. It is worth noticing that there was only 5.1% grade 3 complications in the JCOG 0804 trial in which majority of the procedures were wedge resection (12). Thus, it would be unwise to consider segmentectomy together with wedge resection with the intention to reduce perioperative risks, especially in high-risk patients when safety is the major concern. For normal-risk patients, it is also necessary to examine the perioperative benefit of segmentectomy separately from that of wedge resection instead of mixing them together as sublobar resections.

In fact, segmentectomy may be technically not less or sometimes even more demanding than lobectomy because of the complexity and variability of hilar structures, precise localization of small lesions, and identification and management of intersegmental planes (18). In the CALGB trial, around 80% of procedures were completed by VATS. However, conversion rates were similar for lobectomy and sublobar resections (6.5% in each arm). There is reason to suspect that conversion rate might be even higher in segmentectomy patients. In the JCOG 0802 trial, which compared specifically segmentectomy with lobectomy, the amount of intraoperative blood loss was even higher in the segmentectomy arm (50 ml) than in the lobectomy arm (44.5 ml, *p* = 0.012) (19), although the difference was not of clinical significance. Again, no difference was found in overall morbidity after the two procedures. However, significantly higher rate of air leak was encountered after segmentectomy (6.5%) than after lobectomy (3.8%, *p* = 0.04), resulting in higher need for chest drain reinsertion (3.8 vs. 1.4%, *p* = 0.015). A previous propensity score-matched study has also reported higher incidence of air leak and pulmonary complications after segmentectomy compared to lobectomy before and after matching (20).

Thus, it is still too early to assume that segmentectomy as a less resection than standard lobectomy carries with it reduced surgical risk for functionally compromised patients, or if it has a perioperative benefit of better recovery as a curative procedure for normal-risk patients. Evidences supporting this notion are still at low levels, such as a meta-analysis showing that the odds ratio for postoperative morbidity after segmentectomy was 0.71 compared to lobectomy (21), but without including results from the CALGB 140503 or the JCOG 0802 trials. Both resection extent and surgical complexity, as well as influence of incision, should be carefully considered. A good example is that in a propensity score-matched study, less severe postoperative complications were noticed after segmentectomy than after lobectomy (OR 0.52, *p* <0.0001) (22). However, the difference between the two groups disappeared after they were matched by the VATS approach. It would be critically important to reconsider the perioperative benefit of segmentectomy separately with that of wedge resection in the context of modern MIS setting.

## Pulmonary Function Preservation With Less Extent of Resection

In addition to oncological prognosis and perioperative outcomes, preserving lung function is also a main consideration in selecting segmentectomy instead of lobectomy for patients with early-stage non-small cell lung cancer (NSCLC). In the LCSG trial (2), sublobar resections preserved higher lung function than lobectomy 06 months after surgery. However, almost 1/3 of the sublobar group had wedge resection. In the single-arm JCOG0804 trial wherein wedge resection was the dominant procedure, postoperative FEV1 and FVC loss after 6 and 12 months was less than 5% (12). Then, there was an impact from surgical incision that was not evaluated in both these two trials. Among patients who underwent VATS lobectomy, segmentectomy, wedge resection, or simple mediastinal procedures without lung resection, Gu et al. (23) found that functional loss from a VATS incision would amount to around 5%, while wedge resection of lung parenchyma would add an additional 1–2% loss in Forced Expiratory Volume in 1 second (FEV1) and Forced Vital Capacity (FVC).

The assumption that sublobar resections would be function-preserving came from the traditional concept of postoperative pulmonary function prediction based on the volume of lung parenchyma resected (24). This was established in the era of lobectomy as the standard procedure for patients with lung cancer. In a recently published prospective observational study, Chen et al. (25) examined observed vs. expected (O/E ratio) spirometry changes according to traditional calculation in VATS lobectomy and segmentectomy patients. Interestingly, the O/E ratio equaled to 1 after lobectomy, indicating that functional loss could be accurately predicted by the volume of parenchyma resection. However, the O/E ratio increased markedly with segmentectomy. The less number of segments removed, the greater the discrepancy between the observed and the expected functional loss in segmentectomy patients. In both studies by Gu et al. (23) and Chen et al. (25), average pulmonary function loss per segment resected in terms of FEV1, FVC, and DLCO were almost doubled after segmentectomy compared with after lobectomy. Thus, it is not surprising that when Chen et al. (25) used number of segments removed/number of segments in the corresponding lobe as the resection index, significant functional preservation was detected only in patients who underwent segmentectomy less than 1/2 of the corresponding lobes. For left upper lingual-sparing segmentectomy, basal segmentectomies of the lower lobes, and combined segmentectomies with resection index greater than 1/2, no significant difference was found in FEV1, FVC, or DLCO changes after surgery compared with corresponding lobectomies. Harada et al. also found a strong association between number of removed segments and loss in FVC after surgery (26). These would also help explain why in the JCOG 0802 trial, functional benefit from segmentectomy over lobectomy was merely 2.7–3.5%, which is much less than the expected criterion of 10% in the study design.

Thus, it seems that less lung parenchyma resection would not necessarily translate to better function preservation, which might be potentially caused by less satisfactory re-expansion of the residual lobe after segmentectomy (23). Another reason might be that the remaining lobe(s) in the ipsilateral or contralateral lung after lobectomy expands and compensates better than after segmentectomy, as suggested by Kim et al. (27). It is important to examine which types of segmentectomy would indeed have a functional-preserving advantage over their corresponding lobectomies, especially in physiologically compromised patients. Of course, saving more lung parenchyma is still a major concern in patients with multiple primary lesions, as they probably would need more than one resection either simultaneously or later.

## Conclusion

With the application of CT screening for lung cancers, there is an increasing need to use less traumatic intervention for both normal-risk and high-risk patients with early-stage disease. Whether segmentectomy as a less resection than lobectomy is oncologically appropriate as a curative procedure should be examined separately according to different types of tumor biology. Indications for sublobar resections should be decided according to current tumor staging and histological classifications, which differentiate GGO-containing lesions from pure solid ones. Surgical risks and functional changes after segmentectomy need to be carefully evaluated according to surgical approaches, resection extent, and complexity of procedures. When we talk about segmentectomy for early-stage lung cancers nowadays, it is important to focus on indolent GGO-containing tumors in a minimally invasive surgery setting, in addition to resection extent, as summarized in Table 1. Future studies should be designed to define the extent of resection more accurately by tumor stage and histology, so patients with lung cancer could benefit from the combination of MIS with less trauma and segmentectomy as a less resection than lobectomy.

**Table 1:** Current evidence and unsolved questions on segmentectomy for ground glass opacity (GGO)-containing early-stage lung cancers.

**Topic**	**Question**	**Current evidence**	**Pending question**
Oncological outcome	Is segmentectomy non-inferior to lobectomy?	JCOG 0802: Yes Total lesion size ≤ 2cm, CTR>0.5	How about: 2 cm < Total lesion size ≤ 3 cm but solid component size ≤ 2 cm?
Perioperative risk	Is segmentectomy better than lobectomy?	JCOG 0802:No CALGB 140503:No Both included thoracotomy patients	How about in the MIS setting?
Pulmonary function preservation	Is segmentectomy better than lobectomy?	LCSG: sublobar better than lobectomy (wedge resection included); JOCG0802: Segmentectomy 2.7%- 3.5% better than Lobectomy, Expected difference not reached	Which segmentectomies may be function preserving?

## Author Contributions

WF contributed to conception and design of the article. HL and CS accomplished the first draft of the manuscript. YC and YW searched and collected relevant articles. CZ wrote a section of the manuscript. All authors contributed to manuscript revision, read, and approved the submitted version.

## Funding

This work was supported by the Shanghai Hospital Development Center (grant no: SHDC2020CR1023B).

## Conflict of Interest

The authors declare that the research was conducted in the absence of any commercial or financial relationships that could be construed as a potential conflict of interest.

## Publisher's Note

All claims expressed in this article are solely those of the authors and do not necessarily represent those of their affiliated organizations, or those of the publisher, the editors and the reviewers. Any product that may be evaluated in this article, or claim that may be made by its manufacturer, is not guaranteed or endorsed by the publisher.
